# Integrated Experimental and Computational Insights into the Systematic Synthesis of Cyclodextrin-based MOF

**DOI:** 10.1021/acsomega.5c12081

**Published:** 2026-02-03

**Authors:** Busra Ipek, Zeynep Pinar Haslak, Bünyemin Cosut, Hande Öztürk, Ilknur Erucar

**Affiliations:** † Department of Natural and Mathematical Sciences, Faculty of Engineering, 155531 Ozyegin University, Cekmekoy, 34794 Istanbul, Türkiye; ‡ Istanbul Technical University, Department of Chemistry, Faculty of Science and Letters, Maslak, 34475 Istanbul, Türkiye; § Department of Chemistry, 52962 Gebze Technical University (GTU), 41400 Gebze, Kocaeli, Türkiye; ∥ Department of Mechanical Engineering, Faculty of Engineering, 155531 Ozyegin University, Cekmekoy, 34794 Istanbul, Türkiye

## Abstract

Edible cyclodextrin-based metal organic frameworks (CD-MOFs) represent a sustainable platform for green synthesis. Yet, their reproducible synthesis remains a challenge for the MOF community. In this work, we explored the reproducibility of CD-MOF synthesis and resulting structural variations through five independent sample sets, each repeated three times via a controlled methanol vapor diffusion method. Increasing reaction temperature from 25 to 50 °C enhanced the thermodynamical favorability of γ-CD-MOF crystallization, as confirmed by Density Functional Theory (DFT) calculations. After identifying the optimized synthesis conditions that produced uniform cubic crystals with a high yield (83%), the resulting γ-CD-MOFs were tested for curcumin adsorption. The maximum loading capacity of curcumin reached 24.6%, with an equilibrium uptake of 1.5 mg/g at low concentrations, consistent with Langmuir monolayer adsorption. DFT calculations and molecular simulations revealed that hydrogen bonding, hydrophobic interactions, and strong host–guest affinity (∼65 kcal/mol) govern the adsorption process, predicting a maximum theoretical uptake capacity of 195.6 mg curcumin/g γ-CD-MOF. These results establish reproducible synthesis parameters and demonstrate the enhanced curcumin loading and stabilization capability of γ-CD-MOFs, underscoring their potential as efficient carriers for bioactive molecules.

## Graphical abstract

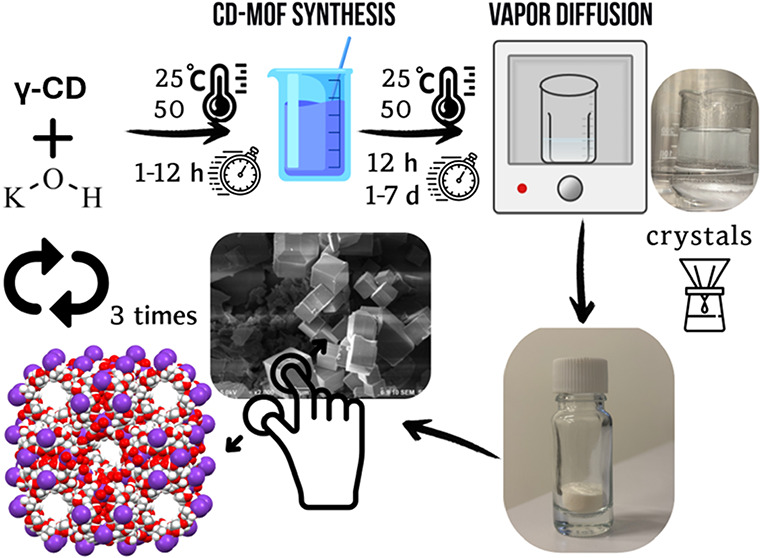

## 1 Introduction

Sugar-based, edible metal organic frameworks (MOFs) represent an emerging class of MOFs due to their sustainability, biodegradability, and natural abundance.^1^ These MOFs are synthesized using cyclodextrins (CDs), cyclic oligosaccharides derived from starch via enzymatic conversion. In 2010, Smaldone et al.^2^ reported the first CD-MOF, synthesized through vapor diffusion of methanol using γ-CD and KOH. This synthesis established a general strategy for developing edible CD-MOFs derived from α-, β-, or γ-CDs with various alkali metal cations.^3–5^ CD-MOFs combine the porous architecture of conventional MOFs with the molecular encapsulation properties of CDs, forming highly ordered frameworks that exhibit enhanced surface area, stability, solubility and bioavailability for guest molecules.^6^ Among CD-MOFs, γ-CD-MOFs offer unique advantages over their α- and β-CD counterparts. The larger cavity size of γ-CDs (9.5 Å versus 5.7 Å for α-CD and 7.8 Å for β-CD) enables the accommodation of bulkier guest molecules, enhancing loading capacity.^6,7^ In addition, the higher symmetry of γ-CD contributes to superior structural stability in γ-CD-MOFs.^8^ The presence of single-bonded functional groups (−OCCO−) on both primary and secondary faces of γ-CD not only improves biocompatibility and nontoxicity but also facilitates efficient formation of complexes with alkali and alkaline earth metal ions, making γ-CD the preferred precursor for CD-MOF synthesis.^9^ Furthermore, γ-CD-MOFs generally exhibit more uniform morphologies due to their regular cubic three-dimensional arrangement, a critical feature for optimizing adsorption amount.^10^

In recent years, CD-MOFs have shown particularly promising potential for the adsorption of pharmaceuticals and bioactive molecules.^6^ Many bioactive compounds exhibit valuable antioxidant, anticarcinogenic, anti-inflammatory and antimicrobial properties, yet their practical application is often limited by poor water solubility.^11^ To overcome these challenges, CD-MOFs have been used to improve the solubility and stability of various biomolecules, including myricetin,^12^ quercetin,^13^ terpinen-4-ol,^7^ menthol,^14^ trans-N-p-coumaroyltyramine,^15^ and resveratrol.^16^ Among these bioactive molecules, curcumin ((1*E*,6*E*)-1,7-bis­(4-hydroxy-3-methoxyphenyl)-1,6-heptadiene-3,5-dione), a polyphenolic compound derived from *Curcuma longa* (turmeric), has garnered interest due to its anti-inflammatory, antimalarial, anticancer, antioxidant and antimicrobial activities.^17^ However, despite its promising therapeutic potential, practical application of curcumin is limited due to its low stability, low bioavailability and poor water solubility, attributed to its hydrophobic nature.^18,19^ To address these issues, various strategies such as encapsulation, protection and controlled release systems have been explored. For example, Chen et al.^20^ demonstrated the successful incorporation of curcumin into γ-CD-MOFs, which not only improves its stability under basic conditions but also achieves higher loading capacities compared to native γ-CD. Moussa et al.^21^ showed that crystallinity of CD-MOF is preserved upon the encapsulation of curcumin, and the stability of curcumin in aqueous and alkaline environments is improved. In another work, Kang et al.^22^ reported that curcumin-loaded cyclodextrin-based metal–organic frameworks (Cur-CD-MOFs), constructed with potassium ions as the metal centers, exhibit strong inhibitory and bactericidal effects. Cur-CD-MOFs also exhibited outstanding aerodynamic performance with increased solubility and dissolution rate of curcumin when Cai et al.^23^ designed a dry powder inhalation formulation of curcumin, which opened new horizons in the delivery of insoluble drugs for pulmonary administration.

Since the initial synthesis of CD-MOFs,^1,2^ numerous synthetic methodologies have been developed, such as vapor diffusion,^24^ hydrothermal/solvothermal,^10,25^ microwave^26^ and ultrasound assisted^27^ methods, to optimize reaction times and control crystal sizes. Each synthesis strategy produces crystals with distinct physicochemical characteristics, tailored to specific functional applications.^24^ Although, edible γ-CD-MOFs offer sustainable solutions in the MOF field, their reproducible synthesis is still challenging. The challenge of reproducibility arises from the fact that many published studies report inconsistent synthesis conditions. To achieve high reproducibility across different synthesis methods, rigorous control of experimental conditions and the adoption of standardized protocols are needed.^28^ In addition, although most groups attempt to validate their synthesis using powder X-ray diffraction (XRD) measurements, they often consider the presence of only two or three Bragg peaks as evidence of a successful synthesis without verifying whether all characteristic peaks expected for CD-MOFs are actually present. Even in studies that include detailed structural analyses, the consistency between different characterization techniques has not been critically examined. Our approach differs by not only addressing the issue of reproducibility but also integrating computational methods to complement experimental characterization results.

In this work, we synthesized fifteen γ-CD-MOF samples under varying reaction and incubation conditions, including temperature and duration, to systematically investigate the crystal growth mechanism. Despite its prolonged synthesis time, we employed the vapor diffusion method with minor modifications, as it offers environmentally friendly and ambient temperature operation, and unlike alternative methods it ensures controlled growth with high product yield,^10,24^ purity^24^ and crystallinity.^1^ Following the synthesis of each γ-CD-MOF, all samples were characterized using powder XRD, Fourier transform infrared spectroscopy (FT-IR), thermal gravimetric analysis (TGA) and scanning electron microscope (SEM) to determine reproducible synthesis conditions. Based on these analyses, we selected optimal conditions for subsequent experiments. Density functional theory (DFT) calculations were performed to understand the crystal growth mechanism under different synthesis conditions. Curcumin, chosen as a model bioactive compound, was then adsorbed into the γ-CD-MOF samples. Finally, molecular simulations were employed to gain deeper insights into the curcumin adsorption behavior of the γ-CD-MOF samples.

## 2 Materials and Methods

### 2.1 Materials

γ-cyclodextrin (γ-CD, purity *>* 99.98%, food grade) was purchased from Wacker Chemicals Co., Ltd. (Shanghai, China). Potassium hydroxide (KOH, purity ≥ 85.0%, pellets) was sourced from Sigma-Aldrich Chemical Co. (St. Louis, MO, USA). Curcumin (C_21_H_20_O_6_, purity ≥ 95.0%) was obtained from AFG Scientific (Northbrook, USA). Methanol (MeOH, purity ≥ 99.8%), potassium chloride (KCl, purity ≥ 99.0%), sodium chloride (NaCl, purity ≥ 99.5%), potassium dihydrogen phosphate (KH_2_PO_4_, purity ≥ 99.5%) and disodium hydrogen phosphate dihydrate (Na_2_HPO_4_·2H_2_O, purity ≥ 99.5%) were supplied by Isolab Chemical Co. (Eschau, Germany). All other reagents used in this study were of analytical grade.

### 2.2 Preparation of γ-CD-MOF

The schematic representation of γ-CD-MOF synthesis is presented in Figure S1 (Supporting Information). γ-CD-MOF crystals were synthesized by a MeOH vapor diffusion method adapted from Smaldone et al.,^2^ with minor modifications to the reported procedure. First, 1.30 g (1 mmol) of γ-CD and 0.45 g (8 mmol) of KOH were weighed and poured into the 50 mL of dry beaker. Then, 20 mL of distilled water (18.2 Ω resistivity, prepared using an Isolab water purification system) was added to the beaker under continuous stirring at 400 rpm using a magnetic stirrer. The reaction beaker was placed inside a larger 600 mL beaker containing 50 mL of MeOH, and the system was sealed with Parafilm to enable vapor diffusion. Following the MeOH vapor diffusion, the resulting γ-CD-MOF crystals were collected by filtration. Then, 30 mL of MeOH was added to wash the crystals, which were then left undisturbed in MeOH for 7 days to remove unreacted components. γ-CD-MOF crystals underwent sequential vacuum drying in an oven (Nüve, EN120 incubator, Ankara, Türkiye) at 25 °C for 10 h and 45 °C for 12 h. Five distinct γ-CD-MOF syntheses were performed by systematically varying reaction parameters, including temperature and incubating time as shown in Table 1. The selection of these conditions was guided by previously reported γ-CD-MOF synthesis protocols in the literature,^2,10,28^ where reaction temperature and incubation time have been shown to influence crystal formation and structural properties. Accordingly, the chosen conditions were defined to enable meaningful comparison of the resulting materials within literature-reported synthesis windows.

**Table 1:** Stirring and Incubation Conditions in γ-CD-MOF Syntheses

Set #	Stirring Temperature and Time	Incubation Temperature and Time
1A, 1B, 1C	25 °C, 12 h	25 °C, 7 d
2A, 2B, 2C	25 °C, 12 h	50 °C, 24 h
3A, 3B, 3C	25 °C, 12 h	50 °C, 12 h
4A, 4B, 4C	50 °C, 1 h	25 °C, 7 d
5A, 5B, 5C	50 °C, 1 h	50 °C, 24 h

For each synthesis, 3 replicate experiments were conducted, with the resulting samples designated as A, B, and C corresponding to their set numbers (e.g., sample 1A represents the first synthesis condition: stirring at 25 °C for 12 h (hour) followed by incubation at 25 °C, 7 d (days). The yield of each γ-CD-MOF sample was calculated as the percent ratio of the obtained product mass to the theoretical mass based on reagent inputs, with all results shown in Table S1.

### 2.3 Curcumin Adsorption

Curcumin was dissolved in absolute ethanol to prepare solutions at varying concentrations (1, 2, 3, 4, and 5 mg/mL). 50 mg of γ-CD-MOF samples were weighed and dispersed into 10 mL of curcumin solution in a glass vial and stirred magnetically in the dark for 24 h at room temperature. The mixture was then centrifuged for 5 min at 3000 rpm. The concentration of curcumin in the supernatant was measured using a single-beam UV–vis spectrophotometer (Shimadzu UV1280, Kyoto, JAPAN) at 426 nm. The adsorption capacity of the γ-CD-MOFs at equilibrium (*Q*_e_, mg/g) was calculated according to eq 1.^29^

1 $$Q_{e}=\frac{{(C}_{o}-C_{e})\times V}{m}$$

Here, *C*_0_ and *C*_e_ (mg/mL) represent the initial concentration of curcumin and the concentration of curcumin at equilibrium, respectively. *V* (mL) denotes the volume of the solution, and *m* (mg) refers to the mass of γ-CD-MOF. In addition, the amount of curcumin adsorbed by γ-CD-MOFs was calculated as a percentage using eq 2.^9^

$$Loading\;Capacity\;\%=\frac{\;Amount\;of\;adsorbed\;curcu\,\min}{Total\;amount\;of\;\gamma ‐CD‐MOF\;and\;curcu\min}\times 100\%$$ 2

The Langmuir adsorption isotherm model^29^ given in eq 3 was applied to further investigate the mechanism of curcumin adsorption onto the synthesized γ-CD-MOFs. This model assumes that the adsorbent surface is homogeneous, with each adsorption site accommodating only one adsorbate molecule. Furthermore, it neglects any interactions between molecules adsorbed at adjacent sites. The UV–vis spectroscopy measurements that were obtained after 24 h adsorption, were used to determine the remaining curcumin amount in the mixtures of γ-CD-MOF and curcumin solution.

3 $$\frac{C_{e}}{Q_{e}}=\frac{1}{K_{L}{\times q}_{m}}+\frac{C_{e}}{q_{m}}$$

In eq 3, *q*_m_ (mg/g) indicates the maximum adsorption capacity, as estimated by the Langmuir isotherm model. The Langmuir constant, denoted as *K*_L_ (L/mg), provides insight into the strength of the interaction between the adsorbent and the adsorbate.

### 2.4 Characterization of the γ-CD-MOFs

#### 2.4.1 Morphology and Crystal Structure

The particle morphology of γ-CD-MOF samples was observed by using Multibeam FIB-SEM system equipped with Oxford Omniprobe Micromanipulator and Gas Injection System (C-GIS), and Oxford Xmax-N EDS System (JEOL JIB 4601F Multibeam SEM-FIB System, Akishima, Tokyo, JAPAN). The samples were adhered to a sample pan by a carbon double-sided tape and sputter-coated with gold by using an automatic sputter coater (CRESSINGTON 108 AUTO, Ted Pella, Inc., Redding, California, USA) twice and then observed and photographed with a working voltage of 5 kV.

The crystallographic structures of γ-CD-MOF samples were determined using X-ray diffraction (XRD) (D8 ADVANCE, Bruker, Karlsruhe, Germany). Cu–Kα X-rays were used during measurements, and the samples were scanned in the 2θ range of 2 to 30°, with a step size of 0.01° and a scan rate of 0.3°/min. Percent crystallinity of each sample was obtained by DIFFRAC.EVA-V7 software (Bruker Corporation, Karlsruhe, Germany).

X-ray photoelectron spectroscopy (XPS) measurements were performed using a monochromatic Al Kα source (*hv* = 1486.6 eV) on a Thermo VG K-α+ instrument (Thermo Scientific, United Kingdom) to evaluate the surface chemical characteristics of the material.

The specific surface area of the γ-CD-MOF was determined by N_2_ adsorption–desorption measurements using a Brunauer, Emmett and Teller (BET) surface area analyzer (NOVA 800, Anton Paar, Graz, Austria). Prior to analysis, approximately 50 mg of the sample was degassed at 60 °C for 8 h. The degassing temperature was selected to avoid structural degradation of the γ-CD-MOF framework. All measurements were carried out at 77 K using liquid nitrogen.

#### 2.4.2 Fourier Transform Infrared Spectroscopy (FT-IR)

The chemical bond and functional groups of γ-CD-MOF, pure curcumin and curcumin adsorbed γ-CD-MOF samples were determined using a Fourier transform infrared spectrophotometer (Shimadzu IRAffinity-1S, Kyoto, JAPAN) equipped with an attenuated total reflectance (ATR) at room temperature. The measurements were carried out a wavenumber range of 600–4000 cm^–1^ at 4 cm^–1^ resolution in 16 scans. Air was used as background in the measurements.

#### 2.4.3 Thermogravimetric Analysis (TGA)

Thermal gravimetric analyses (TGA) of γ-CD-MOF samples and their curcumin loaded samples were taken using a Mettler Toledo Stare Thermal Analysis System from the USA. These analyses were done at a heating rate of 10 °C per minute across a temperature range of 25–800 °C, under a nitrogen flow of 50 mL per minute.

### 2.5 Density Functional Theory (DFT) Calculations

To reduce computational cost, a finite cluster model consisting of one γ-CD layer coordinated by two K^+^ ions was extracted from the experimental crystal structure^2^ and used to represent the γ-CD-MOF framework, as shown in Figure S2. This cluster model carries a net charge of +2, consistent with the coordination environment of the selected γ-CD-MOF fragment. All geometry optimizations and harmonic frequency calculations of the cluster model were performed using the Gaussian 16 program.^30^ Convergence criteria for optimizations were set to default Gaussian conditions, and all optimized structures were confirmed as true minima by the absence of imaginary frequencies.

For the benchmark study of the DFT method, the hybrid-generalized gradient approximation (GGA) exchange-correlation Becke, 3-parameter, Lee–Yang–Parr (B3LYP)^31–33^ functional and the GGA Perdew, Burke and Ernzerhof (PBE)^34,35^ functional, with/without Grimme’s D2 correction,^36^ together with 6-31+G­(d) basis set were tested. Water environment was mimicked by employing the conductor-like polarizable continuum model (CPMC)^37^ and the universal solvent model (SMD).^38^ The selected key bond lengths, namely potassium–oxygen coordination bond (K—O), α-1,4-glycosidic bonds (C—O) and ether functional group in glucose unit (C—O), were compared with the corresponding bond lengths in the crystal structure of γ-CD—K.^2^ The selection of the final computational protocol was based on the closest agreement with experimental structural data and is discussed in detail in the Supporting Information (Table S1).

Using the optimal level of theory determined from the benchmark study (B3LYP-D2/6-31+G­(d) with CPCM solvation), the Gibbs free energy and enthalpy of γ-CD-MOF were calculated in a methanol environment over a temperature range of 15–60 °C. Thermodynamic quantities were derived from frequency calculations using standard statistical thermodynamics within the harmonic approximation.

The vibrational bands of γ-CD-MOF were validated by frequency calculations followed by geometry optimization of the cluster model with B3LYP functional and Grimme’s dispersion correction with Becke-Johnson Damping (D3-BJ)^39^ using split valence polarization Def2-SVP^40,41^ basis set, which were scaled by a factor of 0.9664 ^42^ to compensate for harmonic effects.

The host–guest interactions between curcumin and γ-CD-MOF, as well as between curcumin and γ-CD, were investigated by optimizing the γ-CD-MOF–curcumin and γ-CD–curcumin complexes at B3LYP-D2/6-31+G­(d) level of theory, using an ethanol environment to mimic the conditions under which the curcumin adsorption experiments were performed. Three initial curcumin orientations were tested to improve conformational sampling. The interaction energies between γ-CD-MOF–curcumin and γ-CD–curcumin were determined using the counterpoise correction method,^43,44^ to account for basis set superposition error (BSSE), according to eq 4.

4 $$\Delta E_{\mathrm{int}}=E_{\mathrm{complex}}-\left(E_{\mathrm{host}}-E_{\mathrm{guest}}\right)+\delta^{\mathrm{BSSE}}$$

It should be noted that this approach represents a zero-temperature, static description and does not explicitly account for entropic effects, thermal motion, solvent dynamics, or long-range periodic interactions of the extended MOF framework. Nevertheless, the adopted methodology provides reliable qualitative and comparative insight into the relative strength and nature of curcumin binding in γ-CD-based host systems.

### 2.6 Molecular Simulations

The atomic coordinates of the γ-CD-MOF structure were obtained from the Cambridge Structural Database (CSD)^45^ using the reference code LAJLAL, corresponding to the empirical composition (C_48_H_80_K_2_O_40_^2+^)*_n_*,2*n*(OH^–^), which yields the extended framework formula C_576_H_960_O_480_(KOH)_24_. In constructing the simulation model, the free and disordered OH^–^ ions present in the crystallographic data were initially removed to generate a bare γ-CD-MOF framework. Since the resulting framework contains 24 potassium ions and is therefore cationic, a total charge of +24 was assigned to the bare γ-CD-MOF structure using charge equilibration method (Qeq).^46^ To restore charge neutrality, 24 OH^–^ ions were inserted into the framework via configurational-bias Monte Carlo (CBMC) simulations; these ions act solely as charge-compensating species and do not imply one-to-one coordination with individual K^+^ ions. This procedure ensures electroneutrality while avoiding artificial constraints on the framework geometry.^47^

The geometry of the isolated OH^–^ ion was first optimized using DFT with a net charge of −1. The DFT calculations utilized the GGA of PBE functional with double-numeric polarized (DND)^48,49^ basis set and incorporated D2 correction scheme proposed by Grimme for van der Waals corrections, to obtain a reliable equilibrium geometry of the OH^–^ ion prior to its use in subsequent CBMC simulations. The calculations employed an effective core potential (ECP) with the following convergence criteria: the SCF tolerance was set to 1 × 10^–5^, with a maximum of 50 iterations permitted. The energy convergence threshold was 2 × 10^–5^ Ha, while the force and displacement convergence criteria were set to 0.004 Ha/Å and 0.005 Å, respectively.

The CBMC simulation consisted of 10^6^ production steps, with a maximum loading attempt limit of 10^5^. To identify the lowest-energy configurations, an annealing cycle was applied, where the temperature was automatically varied between 10^5^ K and 100 K over 10 cycles. Geometry optimization at each step was performed using the Smart algorithm, with convergence thresholds set to 500 maximum iterations, an energy tolerance of 10^–3^ kcal/mol, and a force threshold of 0.5 kcal/mol/Å. Interatomic interactions were modeled using the Universal Force Field (UFF),^50^ with Lennard-Jones 12–6 potentials calculated via an atom-based approach and a 12.5 Å cutoff. Electrostatic interactions were treated using the Ewald method with an accuracy of 10^–3^ kcal/mol. The resulting charge-neutral γ-CD-MOF structure is shown in Figure S3.

We finally examined the curcumin adsorption in γ-CD-MOF structure. The geometry optimizations of the keto and enol tautomers of curcumin molecule were performed at the B3LYP-D2/6-31+G­(d,p) level using the Gaussian16 program. Implicit solvation by ethanol using CPCM model was considered to mimic the solvent environment. Since the enol form of curcumin was found to be 11.2 kcal/mol more stable than its keto form, we used the former for molecular simulations. Electrostatic potentials were calculated for the conformer corresponding to local minima using the Merz–Singh–Kollman (MK) scheme (IOp: (6/33 = 2,6/42 = 6,6/50 = 1)) at the HF/6-31+G­(d)^51–53^ level. The RESP (Restrained Electrostatic Potential) fitting was then applied to the electrostatic potentials using the antechamber module provided in the AMBER simulation package^54,55^ to obtain the partial atomic charges in each molecule. The calculated RESP charges are given in Figure S4.

To compute curcumin loading in γ-CD-MOF, CBMC simulations were performed using the Universal Force Field (UFF) with Lennard-Jones 12–6 potentials, employing an atom-based approach and a cutoff distance of 12.5 Å using the Sorption module of Materials Studio 2024 program package.^56^ Electrostatic interactions were modeled using the Ewald method with an accuracy of 10^–3^ kcal/mol. To determine the maximum curcumin loading capacity, sequential CBMC simulations were performed at 298 K by incrementally adding individual curcumin molecules to the system. For CBMC simulations, five types of trial moves were considered, including exchange, regrow, twist, rotation and translation. In each simulation step, both curcumin and 24 OH^–^ ions were introduced simultaneously to maintain charge balance. 10^6^ production and 10^5^ equilibration steps with a maximum loading attempt limit of 10^5^ were used in simulations.

The γ-CD-MOF framework was assumed to be defect-free and structurally rigid, as derived from the experimental crystal structure, and framework flexibility was not explicitly considered during curcumin adsorption to reduce the computational cost. Curcumin adsorption was modeled under equilibrium conditions without inclusion of explicit solvent molecules. In addition, the simulations did not capture kinetic barriers and diffusion pathways. These assumptions represent common approximations in host–guest modeling of MOF systems and allow for a focused evaluation of dominant host–guest interaction energies and loading behavior.

## 3 Results and Discussion

### 3.1 Structural Characterization of the Synthesized γ-CD-MOF Samples

We evaluated the success of crystallization of our 15 synthesized γ-CD-MOF samples based on XRD measurements. According to previous studies,^9,14^ the highest-intensity, characteristic Bragg peaks of γ-CD-MOF are expected to appear around the angles (2θ) of 4.0, 5.6, 6.9, 8.0, 9.0, 9.9, 13.3, and 16.6°. These Bragg angles match well with those computed from forward simulation of powder diffraction data by VESTA.^57^ Figure 1 presents the XRD measurements obtained from samples 1ABC (a), 2ABC (b), 3ABC (c), 4ABC (d) and 5ABC (e) along with the expected Bragg angles indicated by red vertical lines.

**Figure 1:**
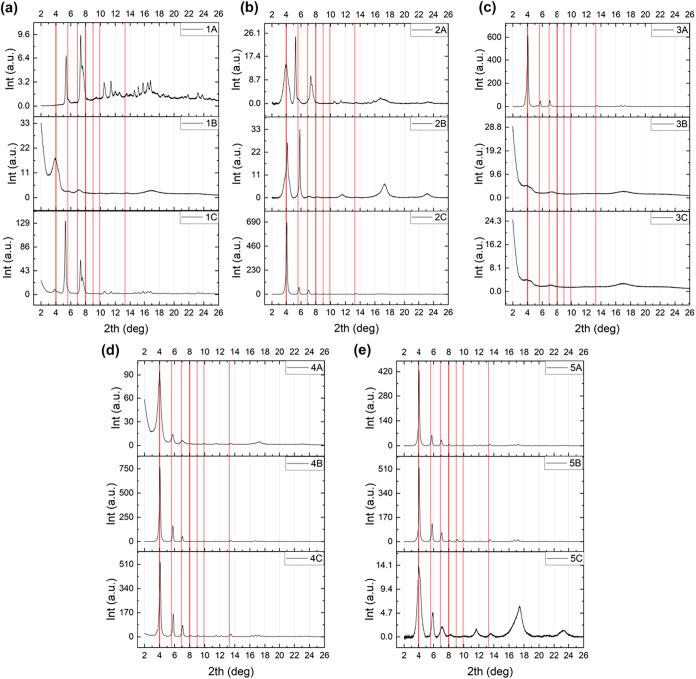
XRD patterns of γ-CD-MOF sample groups, (a) 1ABC, (b) 2ABC, (c) 3ABC, (d) 4ABC, and (e) 5ABC.

Sample 1A exhibited crystalline features; however, none of the observed reflections matched the characteristic peaks of γ -CD-MOF, suggesting the formation of an unidentified crystalline phase. Apparent from the lack of well-defined peaks, 1B showed almost no crystallization. Although sample 1C showed evidence of crystallinity, similar to 1A, its diffraction peaks did not align with the expected Bragg peak positions of γ-CD-MOF. Collectively, these results suggested that the synthesis conditions employed for these samples were not optimized for the formation of high-quality γ-CD-MOF crystals.

The XRD patterns of sample set 2 indicated a higher degree of crystallization compared to set 1. All three samples (2A, 2B, and 2C) exhibited clearly identifiable Bragg reflections, with the first peak appearing at the expected Bragg position. However, for 2A and 2B, the remaining peaks were either shifted from their expected positions or absent altogether, suggesting suboptimal crystallization. In contrast, sample 2C displayed its first three Bragg peaks at the expected positions, with sharp peak profiles, indicative of successful crystallization. Within sample set 3, only 3A exhibited a diffraction pattern consistent with high-quality γ-CD-MOF. Overall, the results from sample sets 1–3 demonstrated poor reproducibility in obtaining phase-pure, well-crystallized γ-CD-MOF.

In comparison, the fourth and fifth synthesis methods yielding sample sets 4 and 5 exhibited better reproducibility than the sample sets 1–3 (see Table S2), in agreement with previous reports.^20,21,58,59^ With the exception of samples 4A and 5C, the diffraction patterns featured sharp, well-resolved peaks with high photon counts, indicative of high-quality crystals with relatively large average crystallite sizes (Figure 1). In contrast, the lower photon counts, and broadened Bragg peaks observed from the diffraction profiles of 4A and 5C suggested substantially smaller average crystallite sizes. For nearly all samples in sets 4 and 5, the diffraction peaks were well aligned with the expected Bragg angles (red vertical lines), except for 5C, which displayed significant deviations and pronounced broadening. As a result, the fourth synthesis method yielded the most favorable and reproducible results among the five methods tested, producing γ-CD-MOF with a consistently high average product yield of 83%.

The structural characterization of γ-CD-MOF-5A sample was further supported by the XPS spectra. XPS spectra in Figure 2 displays the chemical and oxidation states, as well as the binding energies, for the C, O, and K elements. The survey spectrum in Figure 2A confirmed the presence of C, O, and K elements, and it indicated the successful formation of the CD-MOF structure. The C 1s spectrum exhibited characteristic peaks corresponding to C–C, C–O, and O–C–O bonds, confirming the preservation of the cyclodextrin framework. The appearance of a lower binding energy component in the O 1s spectrum was attributed to K–O coordination, indicating the interaction between potassium ions and cyclodextrin hydroxyl groups. The K 2p spectrum displayed characteristic doublet peaks corresponding to K^+^ species, confirming the incorporation of potassium ions into the CD-based framework. Therefore, all spectra indicated that the cyclodextrin framework was preserved and the construction of the CD-MOF was confirmed.

**Figure 2:**
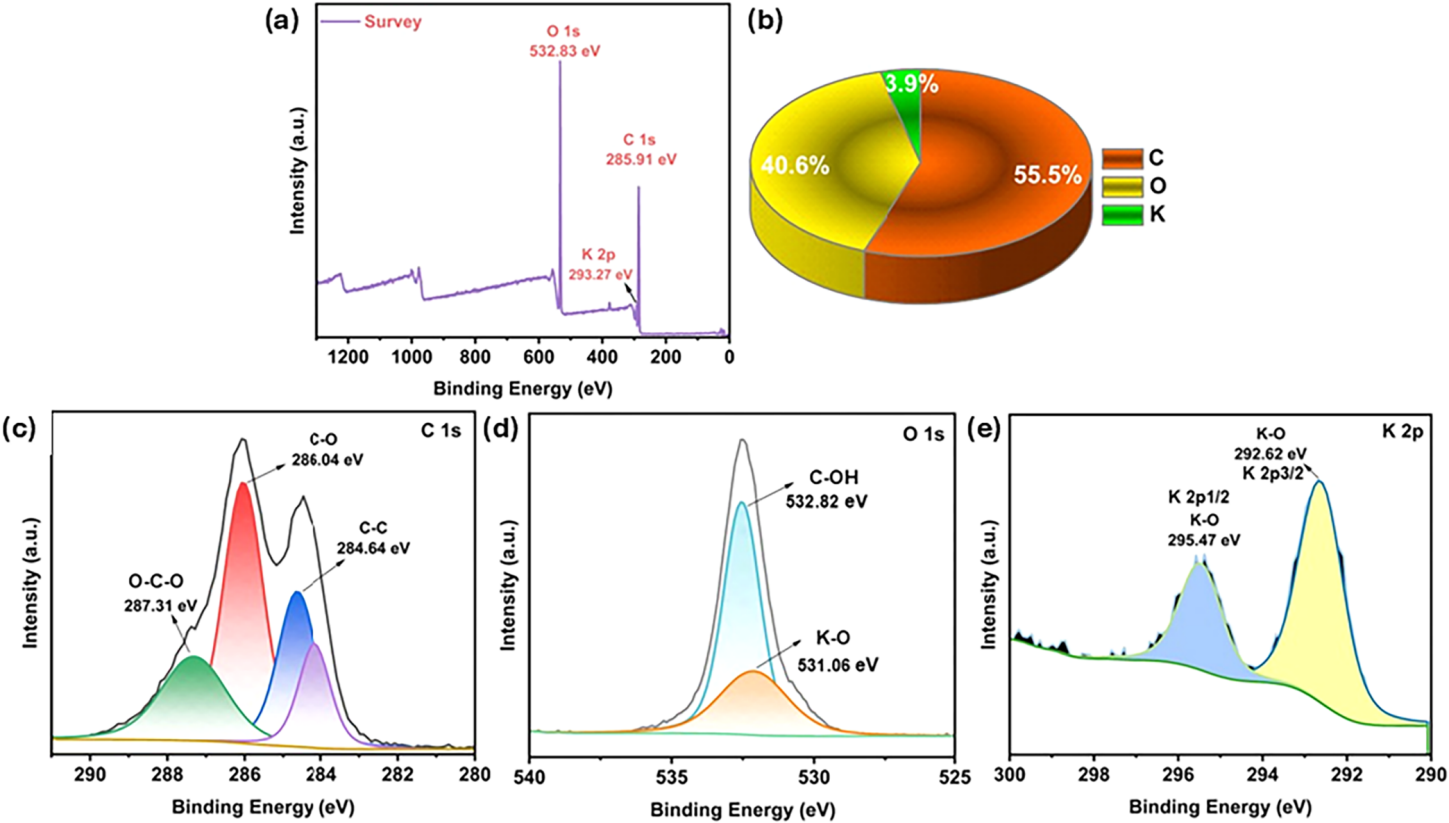
XPS spectra (a) survey, Elemental percentage pie graph (b), C 1s (c), O 1s (d), K 2p (e) of CD-MOF.

The morphology and surface characteristics of the synthesized γ-CD-MOFs were examined by scanning electron microscopy (SEM). As shown in the SEM images (Figure 3), the γ-CD-MOF samples exhibited distinct morphological differences that correlated closely with the synthesis conditions: Samples 1A and 3A predominantly exhibited plate-like particles with broad size distributions, complementing the moderate crystallinity. Sample 2A consisted mainly of small, irregularly shaped particles with rough surfaces, indicative of less controlled crystal growth. In contrast, sample 4A displayed large, uniform cubic crystals with sharp facets and smooth surfaces, consistent with a more controlled and uniform growth process. This particular morphology of sample 4A was consistent with the typical γ-CD-MOF morphology reported in the literature (see Figure 1B in ref 60).^2,58,60^ Sample 5A also exhibited plate-like particles, large in size and indicative of high-quality crystallization. Interestingly, the relatively large crystallite size of 4A shown in Figure 3 did not align with the corresponding XRD data (Figure 1). The diffraction peaks of 4A exhibited greater broadening than those of 3A and 5A, indicating smaller average crystallite sizes for 4A compared to 3A and 5A. This discrepancy suggested two possible explanations: either the cubic particles of 4A observed in Figure 3 were polycrystalline rather than single crystalline, or the XRD measurement for 4A predominantly sampled much smaller crystallites, thereby failing to capture structural information from the larger ones. Overall, the SEM observations underscored the significant influence of synthesis conditions on particle morphology, with method 4 producing the most distinct and well-defined crystal morphologies.

**Figure 3:**
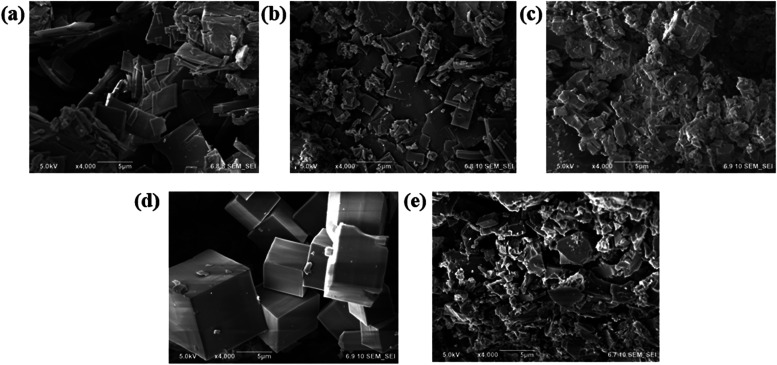
SEM images of γ-CD-MOF samples (a) 1A, (b) 2A, (c) 3A, (d) 4A, and (e) 5A.

Finally, the average particle and crystallite sizes were obtained through analysis of the SEM and XRD data sets. The SEM-derived particle sizes represented the average dimensions of visibly isolated particles in Figure 3 and were measured by processing the SEM images using ImageJ^61–63^ (Table 2). In contrast, the XRD-derived crystallite sizes correspond to the average dimensions of crystalline domains; coherent, uninterrupted subvolumes of the three-dimensional periodic atomic structure within the particles. These values were calculated by fitting a Pseudo-Voigt function to the first three diffraction peaks of each sample. The full width at half-maximum (FWHM) of the peaks was then related to the average crystallite size using the Scherrer equation,^64^ and the final average crystallite size was obtained by averaging the values derived from the three diffraction peaks.

**Table 2:** Average Particle and Crystallite Sizes of the Synthesized γ-CD-MOF Samples^a^

Sample Name	Particle Size (SEM, μm)	Crystallite Size (XRD, μm)
1A	3.626 ± 1.293	0.43
2A	1.570 ± 0.865	0.39
3A	1.324 ± 0.767	0.50
4A	5.204 ± 2.894	0.22
5A	6.785 ± 2.222	0.47

a SEM-based values were obtained from a minimum of 62 crystals.

According to Table 2, SEM-based average particle sizes were in the order of a few micrometers and particle sizes of 1A, 2A, and 3A samples were slightly less than those of 4A and 5A. However, due to the relatively high statistical uncertainties, the difference between particle sizes of different samples was unimportant. In comparison, XRD-based crystallite sizes were all on the order of hundreds of nanometers and they were below SEM-based sizes. This was expected due to the different definitions of size in each technique. The large difference between the particle and crystallite size suggested that the isolated particles identified from SEM images were likely polycrystalline, or there were imperfections in the microstructure.

To evaluate the effect of temperature on the relative stability of γ-CD-MOF in methanol environment, thermodynamic parameters, including Gibbs free energy and enthalpy, were calculated at temperatures between 15 and 60 °C by performing DFT calculations (Cartesian coordinates for the γ-CD-MOF cluster model are given in Table S3). As shown in Figure 4, which presents the temperature dependence of relative Δ*G* and Δ*H* with respect to the γ-CD-MOF structure at 15 °C, increasing temperature led to more negative Δ*G* values, indicating that crystallization becomes thermodynamically more favorable. In contrast, Δ*H* increased with temperature, suggesting that the enthalpic contribution is progressively unfavorable. Thus, the stabilization of γ-CD-MOF at elevated temperatures was attributed to an increasingly favorable entropic contribution, which points out that at higher incubation temperatures more organized crystalline structures are attained.

**Figure 4:**
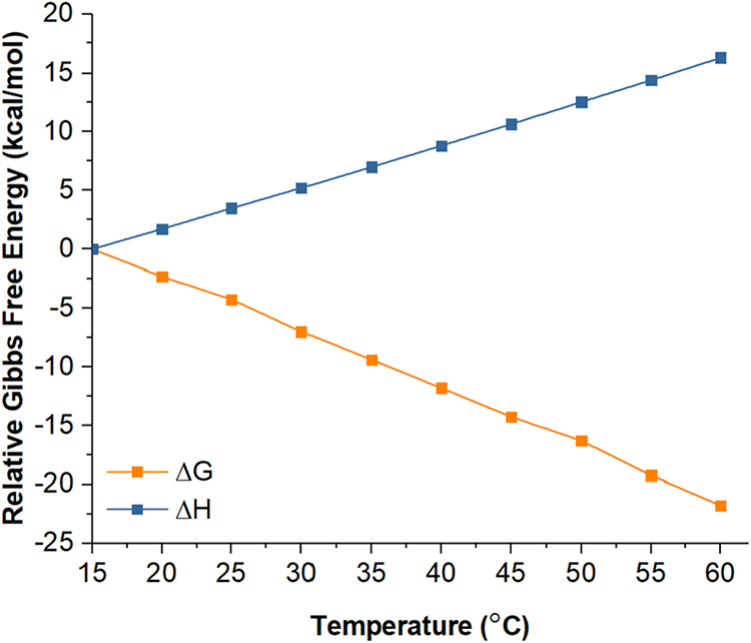
Plot of the thermodynamic parameters Δ*G* and Δ*H* for the γ-CD-MOF cluster model as a function of temperature calculated at B3LYP-D2/6-31+G­(d) level using CPCM solvation model and methanol as solvent. Δ*G* and Δ*H* values refer to the relative Gibbs free energy and enthalpy values calculated at 15 °C.

These observations align with the findings of Liu et al.,^28^ who reported that increasing the synthesis temperature promotes vapor diffusion of methanol into the reactant solution, leading to rapid supersaturation of the precursors. This effect facilitates the formation of more uniform and cubic γ-CD-MOF crystals in a shorter synthesis time without significantly compromising crystallinity. Similarly, this observation parallels the results of Hamedi et al.,^65^ who demonstrated that well-defined cubic γ-CD-MOF crystals (10–15 μm edge length) were obtained only via a rapid, surfactant-free, temperature-assisted synthesis route, underscoring the necessity of sufficient thermal energy for achieving high-quality crystal morphology. Taken together, these findings indicate that the irregular, fragmented, and compactly stacked crystal structures observed in samples 1A, 2A, and 3A can be attributed to insufficient synthesis temperatures. Additionally, as our SEM results showed that uncontrolled rapid nucleation and growth at elevated temperatures could also result in defective or fragmented morphologies. Overall, the results clearly demonstrated that both the duration and temperature of methanol diffusion played a decisive role in determining the crystallinity and morphology of γ-CD-MOFs. By carefully optimizing these synthesis parameters, it becomes possible to produce γ-CD-MOF structures with tailored properties suitable for various applications such as adsorption.

### 3.2 Curcumin Adsorption

#### 3.2.1 Structural Characterization of the Curcumin Loaded γ-CD-MOF Samples

To identify possible structural changes resulting from curcumin loading, 4 and 5 mg/mL curcumin-adsorbed γ-CD-MOF-5A samples (Cur-5A-4 and Cur-5A-5) were characterized by XRD. As seen in Figure 5, the Bragg peaks were well-separated and sharp in sample 5A-4. Here all peaks were positioned very close to the red lines which coincided with the analytically obtained Bragg angles of the pure γ-CD-MOF samples. Even the four very close Bragg peaks between 16 and 18° were clearly resolved, indicating that the crystalline structure was preserved at a curcumin loading of 4 mg/mL. In contrast, sample 5A-5, loaded with 5 mg/mL of curcumin, exhibited reduced crystallinity. The curcumin diffraction peaks dissipated, indicating molecular-level incorporation, likely due to transformation from a crystalline to amorphous state through interactions with γ-CD-MOFs. The loss of crystallinity may result from excessive curcumin loading within the MOF pores or from curcumin nanoclusters attached to the MOF surface. These findings are consistent with Kang et al.,^22^ who reported that the addition of curcumin to γ-CD-MOFs broadened the XRD peaks (around 22°) and caused three separate diffraction planes to merge into a single peak. XRD measurements on curcumin loaded cyclodextrin samples are also displayed in Figure 5b for comparison.

**Figure 5:**
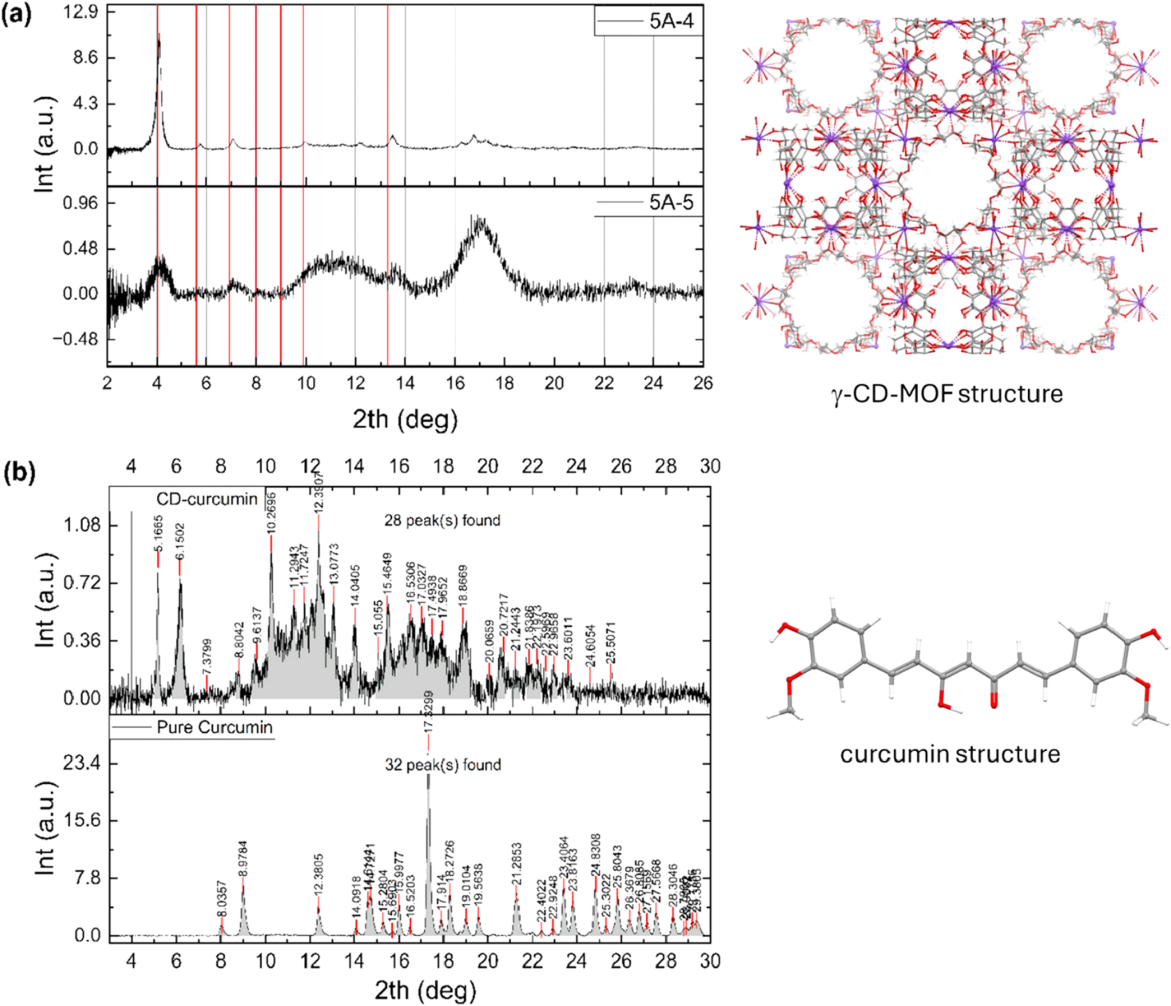
X-ray diffraction data measured from (a) curcumin loaded γ-CD-MOF-5A samples, (b) γ-CD and curcumin. The red lines indicate the positions of the Bragg peaks belonging to pure γ-CD-MOF obtained by analytical calculations. 3D representations of γ-CD-MOF and curcumin structures are displayed on the right.

The FT-IR spectra of the synthesized γ-CD-MOF-5 samples (A–C), together with calculated vibrational bands at DFT level, and 4 and 5 mg/mL curcumin loaded γ-CD-MOF-5A samples (Cur-5A-4 and Cur-5A-5) are presented in Figure 6. A broad absorption band at 3310 cm^–1^ in the synthesized γ-CD-MOF-5 samples was assigned to the O–H stretching vibrations of hydroxyl groups in the glucose units of the γ-CD-MOF framework^66^ (Figure 6a) which corresponded to the sharp peaks between 3700 and 3257 cm^–1^ in the calculated IR spectrum. The peak located at 2922 cm^–1^ arose from the asymmetric C–H and CH_2_ stretching vibrations, a characteristic feature of the γ-CD-MOF structure.^66,67^ These peaks showed no significant variation among three samples, confirming consistent and successful γ-CD-MOF formation. Additionally, −CH and −OH bending vibrations were identified by the peaks at 1352 and 1153 cm^–1^, respectively, in both experimental and calculated spectra. The peak at 862 cm^–1^ was attributed to C–C stretching vibrations. The asymmetric stretching vibrations of C–O–C bonds appeared at 1078 cm^–1^ and at 1018 cm^–1^ experimentally,^66^ which corresponded to sharp calculated peaks at 1100 and 1039 cm^–1^. On the other hand, the peak observed at 1641 cm^–1^ in the spectra of the synthesized samples was not detected in the calculated spectra, suggesting the presence of adsorbed or coordinated water in the samples, as this band is characteristic of H–O–H bending vibrations.^68^

**Figure 6:**
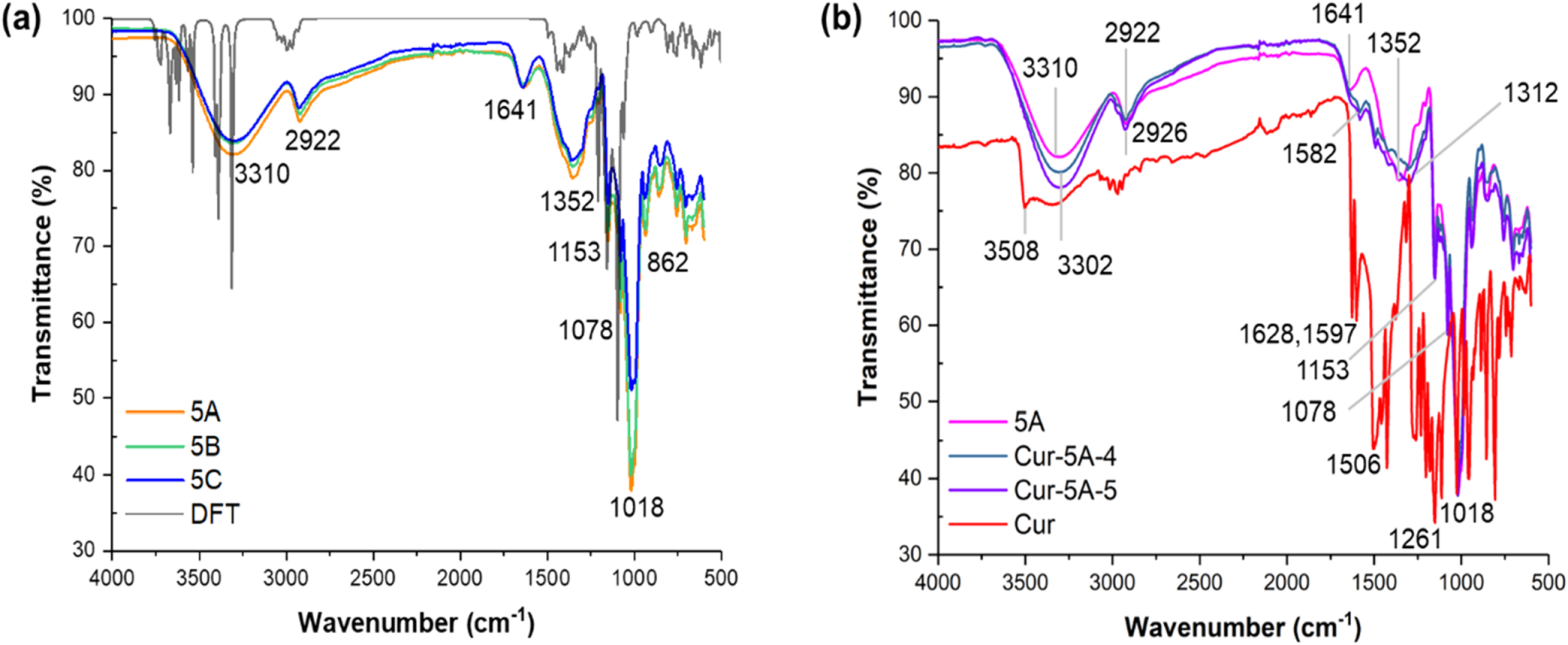
FT-IR Spectra of the sample groups (a) γ-CD-MOF-5 and (b) Cur-5A with the comparison of pure γ-CD-MOF-5A and curcumin samples.

Curcumin-adsorbed γ-CD-MOF-5A samples, prepared using ethanolic curcumin solutions at concentrations ranging from 1 to 5 mg/mL, were also analyzed via ATR-FT-IR spectroscopy to investigate the interactions between curcumin and γ-CD-MOF. As shown in Figure 6b, the spectrum of 4 and 5 mg/mL curcumin-loaded 5A samples (Cur-5A-4 line in green and Cur-5A-5 line in purple) revealed a shift of a major peak from 3310 cm^–1^ in pristine γ-CD-MOF (line in pink) to 3302 cm^–1^ in the Cur-5A samples. Additionally, the characteristic phenolic vibration peak of curcumin at 3508 cm^–1^ was absent in the Cur-5A spectrum. These observations suggested the formation of hydrogen bonds between the hydroxyl groups of curcumin and the −OH groups of the cyclodextrin moieties in the CD-MOF, consistent with the previously reported findings.^21,69^ More substantial shifts from 1352 to 1312 cm^–1^ which corresponded to C–H bending vibrations, further confirmed strong hydrophobic interactions and van der Waals forces between curcumin and the γ-CD-MOF framework.^20^ On the other hand, mixed C=C and C=O stretching vibrations, C=C stretching vibrations of benzene ring, C=O and C–O stretching of curcumin which appeared at 1628 cm^–1^,^70^ 1597 cm^–1^,^21,71^ 1506 cm^–1^,^1,72^ and 1261 cm^–1^ ^71^ were observed to be masked by the overlapping peaks of CD-MOF in the Cur-5A samples.

The host–guest interactions were investigated using DFT calculations on the γ-CD-MOF–curcumin complex. The interaction motifs between curcumin and γ-CD-MOF, shown in Figure S5a–c, revealed that the oxygen atoms of the phenolic methoxy and hydroxy groups of curcumin form hydrogen bonds with the hydroxyl hydrogens of γ-CD-MOF, resulting in an interaction energy of −39.0 kcal/mol (Figure S5a). This finding corroborated the hydrogen-bond formation observed in the FT-IR spectra. In addition, ion–dipole interactions between the potassium ion and the phenolic hydroxyl oxygen atom of curcumin yielded a stronger interaction energy of −55.2 kcal/mol (Figure S5b). When both hydrogen bonding and ion–dipole interactions coexist between γ-CD-MOF and curcumin, a markedly enhanced interaction environment is obtained, with an interaction energy of −64.3 kcal/mol (Figure S5c). In contrast, the interaction of curcumin with γ-CD alone is governed exclusively by hydrogen bonding between the hydroxyl groups of γ-CD and those of curcumin, as well as the carbonyl oxygen of curcumin, corresponding to an interaction energy of −48.7 kcal/mol (Figure S5d). Overall, these results demonstrated that γ-CD-MOF exhibits superior encapsulation capability for curcumin compared to γ-CD, which can be attributed to the strong ion–dipole interactions between the coordinating K^+^ ions in the MOF framework and the oxygen atoms of curcumin.

To further evaluate the reproducibility and thermal stability of γ-CD-MOF-5 samples, sample 5C was selected, and the same methodology described above was used for curcumin loading. The resulting thermogravimetric curves of γ-CD-MOF-5 and its curcumin loaded counterparts (4 and 5 mg/mL) are presented in Figure 7. All samples exhibited a characteristic two-step weight loss behavior. The initial weight loss below approximately 120 °C corresponded to the removal of adsorbed and coordinated water molecules. The major decomposition step occurred between 300 and 400 °C, which can be attributed to the degradation of the γ-cyclodextrin organic framework. The final residue above 600 °C was consistent with the formation of potassium carbonate species, in agreement with literature reports for γ-CD-MOF materials.^2^ The similar decomposition profiles confirmed that the modified synthesis route yielded a structurally analogous material with comparable thermal stability.

**Figure 7:**
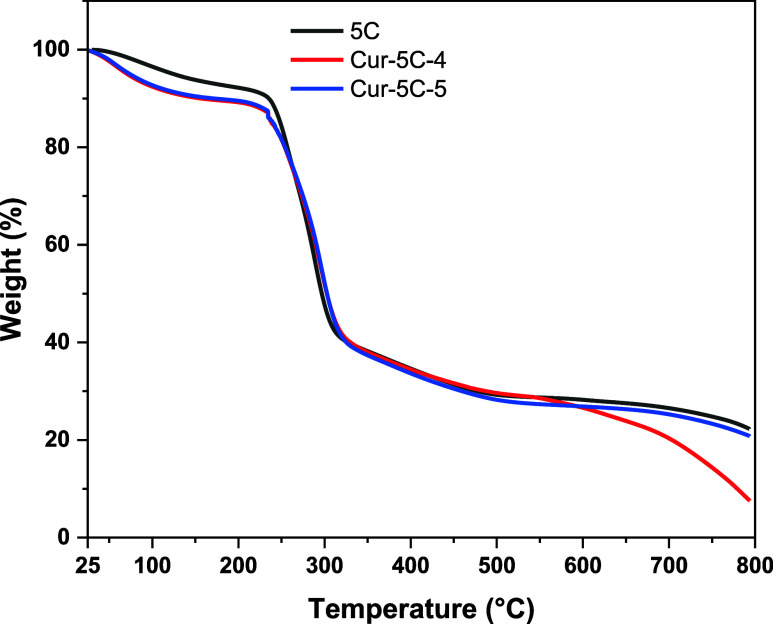
Thermogravimetric curves of γ-CD-MOF-5C and its curcumin loaded counterparts (4 and 5 mg/mL).

#### 3.2.2 Adsorption of Curcumin in γ-CD-MOF Samples

The adsorption capacity of synthesized γ-CD-MOF-5 samples was then examined at low curcumin concentrations ranging from 0.001 to 0.016 mg/mL by using a single-beam UV–vis spectrophotometer and eq 1, as displayed in Figure 8a. Accordingly, this concentration interval was selected to coincide with the established calibration range of curcumin, thereby ensuring reliable quantification of the adsorbed amount. Since the linear correlation between absorbance and concentration is generally preserved only at low concentrations, calibration was carried out within this range. Samples exceeding the calibration interval were therefore diluted to fall within the linear domain, and their actual concentrations were subsequently determined by correcting with the appropriate dilution factor.^73^ As can be seen in Figure 8a, the amount of adsorbed curcumin in three γ-CD-MOF-5 samples continuously increased as the curcumin concentration was increased to 0.012 mg/mL. At this concentration, γ-CD-MOF-5 samples reached to a saturation point, whereafter only a slight increase in the amount of adsorbed curcumin was detected, for which the maximum adsorption was determined to be 1.46 mg of curcumin per gram of γ-CD-MOF.

**Figure 8:**
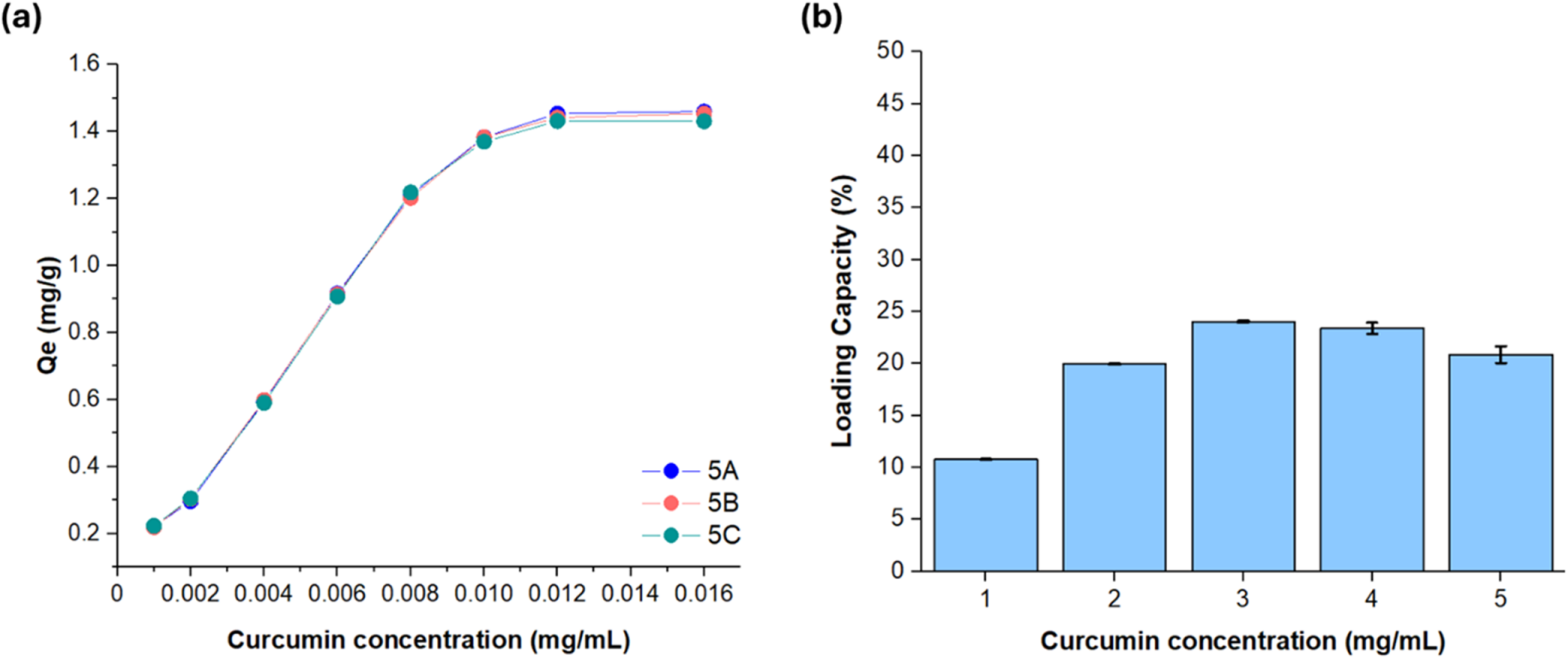
Curcumin (a) adsorption capacities of synthesized γ-CD-MOF samples 5A, 5B, and 5C at low concentration and (b) loading capacities at high concentration.

The loading capacity of curcumin within the γ-CD-MOF samples was investigated using higher concentrations of curcumin (between 1–5 mg/mL) as shown in Figure 8b. As the concentration of the curcumin solution increased from 1 to 2 mg/mL, a significant rise in loading capacity was observed from 10.78 ± 0.06% to 19.92 ± 0.16%. Further increasing the concentration to 3 mg/mL resulted in peak loading capacity of 24.59 ± 0.60%, in harmony with the previous studies.^22^ These results confirmed that higher curcumin concentrations enhance adsorption, likely due to stronger interactions between curcumin molecules and the pores of the γ-CD-MOFs. However, when the concentration reached 5 mg/mL, a slight decline in loading capacity occurred, dropping to 20.86 ± 0.81%. This suggested that the γ-CD-MOFs reached their adsorption limit, and the excess curcumin could no longer be accommodated within the pores.

Curcumin was also loaded into γ-CD, affording a loading capacity of 7.05%, which was consistent with the reported values in the literature.^18,20,22^ Yet, both were markedly lower than the capacities observed for γ-CD-MOF in this work, indicating that conversion of γ-CD to γ-CD-MOF substantially enhances curcumin loading. The low concentration curcumin adsorption performances of three γ-CD-MOF-5 samples were further investigated based on the Langmuir model. The maximum adsorption capacity, *q*_m_, and Langmuir constant, *K*_L_, were calculated by using eq 3 and presented in Table 3, and the fitting results of Langmuir isotherm was displayed in Figure 9. Three γ-CD-MOF samples showed an excellent fit to the Langmuir isotherm with correlation coefficient (*R* ^2^) values of 0.996, 0.997, and 0.998 for samples 5A, 5B, and 5C, respectively, which supported the assumption of monolayer adsorption on a homogeneous surface with finite, identical sites. Three q_m_ values were very similar across all three samples (1.48–1.53 mg/g), conforming well with the adsorption capacity analysis at low concentration (Figure 8a). These results indicated that the surface area and active sites of three synthesized samples were consistent, and the synthesis method was reproducible. Calculated *K*_L_ values were found to be slightly different from each other, γ-CD-MOF-5C possessing the highest value (11.25 L/mg), which pointed out that its adsorption sites were more energetically favorable, implying stronger interaction between the adsorbate and the surface. Calculated *q*_m_ values based on the Langmuir model fitted at high curcumin concentrations were also found to conform well with the experimentally determined average maximum adsorption capacity (245.92 mg/g) as given in Table 3.

**Figure 9:**
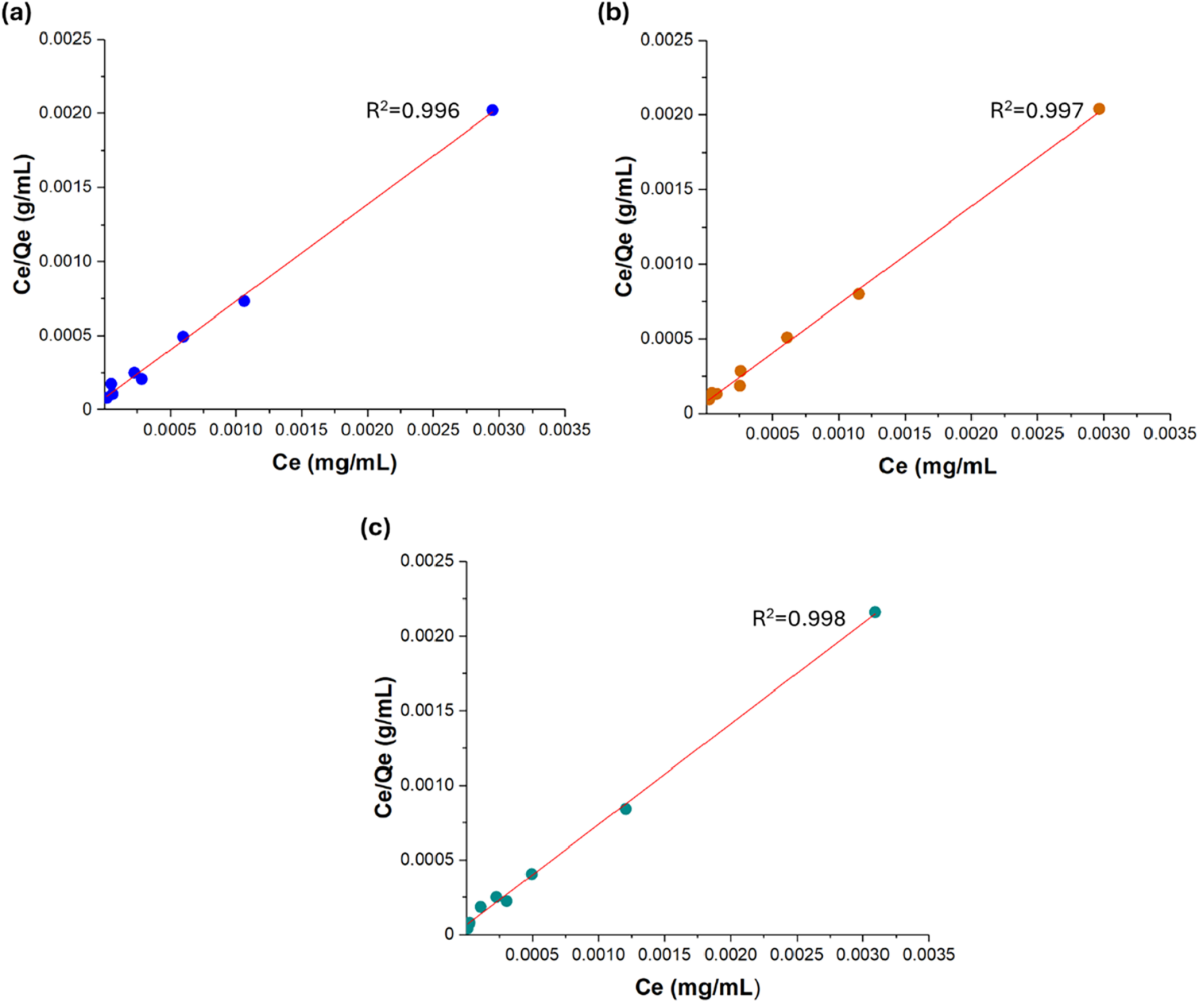
Langmuir isotherms for curcumin adsorption at low concentration for γ -CD-MOF (a) 5A, (b) 5B, and (c) 5C samples.

**Table 3:** Langmuir Isotherm Parameters for Curcumin Adsorption in γ-CD-MOF-5A, γ-CD-MOF-5B, and γ-CD-MOF-5C Samples Loaded with Low (0.001-0.016 mg/mL) and High (1-5 mg/mL) Initial Curcumin Concentrations

γ-CD-MOF samples loaded with low concentration of curcumin	*q*_m_ (mg/g)	*K*_L_ (L/mg)	*R* ^2^
5A	1.53	8.17	0.996
5B	1.52	6.57	0.997
5C	1.48	11.25	0.998

γ-CD-MOF samples loaded with high concentration of curcumin	*q*_m_ (mg/g)	*K*_L_ (L/mg)	*R* ^2^
5A	250.00	0.400	0.999
5B	256.41	0.195	0.999
5C	256.41	0.380	0.999

The validity of the single-layer adsorption assumption was assessed by correlating the specific surface area of the γ-CD-MOF with the molecular dimensions of curcumin. Notably, previously reported γ-cyclodextrin–based MOFs exhibit a wide range of BET surface areas, typically spanning from approximately 300 m^2^/g to values exceeding 1000 m^2^/g, depending strongly on synthesis protocols and activation conditions.^12^ Within this broad range, the BET surface area obtained in the present study (430.8 m^2^/g) was well within expected values, indicating successful framework formation and activation.

Using this surface area together with the reported molecular cross-sectional area of curcumin, the theoretically estimated monolayer adsorption capacity was found to be on the same order of magnitude as the experimentally determined Langmuir maximum adsorption capacity (∼250 mg/g). This close agreement provided strong support for the applicability of the Langmuir model and suggested that curcumin adsorption onto γ-CD-MOF proceeds predominantly via a monolayer coverage mechanism. Moreover, the adsorption performance observed in this work was consistent with previously reported curcumin uptake capacities for γ-cyclodextrin-based MOF systems.^20,21^ Taken together, these results demonstrated that the adsorption capacity achieved herein is not only physically reasonable based on surface area considerations but also in good agreement with existing literature, further validating the robustness of the adsorption behavior of γ-CD-MOF toward curcumin.

Single-component CBMC simulations at the atomic level revealed that at 298 K, γ-CD-MOF demonstrated a maximum storage capacity of ∼12 curcumin molecules per unit cell (corresponding to 195.61 mg curcumin per gram MOF), slightly lower than the experimentally calculated capacity. The discrepancy between the molecular simulation result (195.6 mg/g) and the experimental value (245.92 mg/g) may arise from the idealized assumptions used in the simulation model, such as a rigid framework, uniform adsorption sites, and the absence of solvent effects. Figure S6 shows the distribution of curcumin molecules within the γ-CD-MOF framework. The average isosteric heat of adsorption was calculated to be 66.14 kcal/mol for curcumin. This significantly higher adsorption energy for curcumin indicated these molecules were retained through particularly strong interactions within the framework structure.

## 4 Conclusions

This study systematically elucidated the influence of synthesis temperature and incubation time on the reproducibility of γ-CD-MOF crystallization via an environmentally benign methanol vapor diffusion route. XRD analyses of 15 samples demonstrated that phase-pure γ-CD-MOFs were attainable only under carefully controlled thermal conditions, while complementary FT-IR and SEM measurements of representative samples confirmed the preservation of framework integrity and well-defined cubic morphologies. Among the examined strategies, method 4 (stirring at 50 °C followed by incubation at 25 °C for 7 days) yielded the most reproducible and uniform crystals, with a product yield of ∼83%, and was thus identified as the optimum synthesis condition. Although method 5 also afforded crystalline frameworks, its reproducibility was compromised by the occurrence of irregular morphologies, probably due to the short incubation time (24 h).

The optimized γ-CD-MOFs exhibited markedly enhanced adsorption toward curcumin, achieving a maximum loading capacity of 24.6% and an equilibrium uptake of 1.5 mg/g, values significantly higher than those of native γ-CD. FT-IR analyses revealed hydrogen bonding and hydrophobic interactions as the primary encapsulation mechanisms. Furthermore, DFT calculations corroborated the thermodynamic stability of γ-CD-MOF formation at high temperatures, while CBMC simulations predicted a maximum theoretical storage of 195.6 mg/g, sustained by strong host–guest interactions. Collectively, these findings establish reproducible synthesis protocols for γ-CD-MOFs and highlight their strong potential as efficient carriers for poorly soluble bioactive molecules, particularly in pharmaceutical applications.

## Supplementary Material





## Notes

Benchmark study for DFT calculations; the calculated yields of γ-CD-MOFs; the schematic representation of the synthesis of γ-CD-MOF; 2D representation of generated cluster model of γ-CD-MOF; atomic structure of γ-CD-MOF; 2D representation of enol form of curcumin molecule and the calculated RESP charges; Interactions of curcumin with γ-CD-MOF and γ-CD; atomic structure of curcumin-loaded γ-CD-MOFs; Cartesian coordinates of γ-CD-MOF cluster model (PDF)
Material Preparation Information File (MPIF)^74^ for 15 synthesized γ-CD-MOFs (ZIP)
