# Supplementary material for: Integrated Experimental and Computational Insights into the Systematic Synthesis of Cyclodextrin-based MOF
Source: ACS Omega. 2026 Feb 3;11(6):10554–67. doi: 10.1021/acsomega.5c12081 (PMC12917697; doi:10.1021/acsomega.5c12081)
Supplement: Supplementary file 1 [file ao5c12081_si_001.pdf]

Supporting Information

for

Integrated Experimental and Computational Insights into the Systematic Synthesis of Cyclodextrin-based MOF

Busra Ipek,[a,b§] Zeynep Pinar Haslak,[a§] Bünyemin Cosut,[c*] Hande Öztürk,[d*] and Ilknur Erucar[a,*]

[a]Department of Natural and Mathematical Sciences, Faculty of Engineering, Ozyegin University, Cekmekoy, 34794, Istanbul, Türkiye. [b]Istanbul Technical University, Department of Chemistry, Faculty of Science and Letters, Maslak, 34475, Istanbul, Türkiye. [c]Department of Chemistry, Gebze Technical University (GTU), 41400, Gebze, Kocaeli, Türkiye. [d]Department of Mechanical Engineering, Faculty of Engineering, Ozyegin University, Cekmekoy, 34794, Istanbul, Türkiye.

***Corresponding authors:** bcosut@gtu.edu.tr **,** hande.ozturk@ozyegin.edu.tr, ilknur.erucar@ozyegin.edu.tr

§These authors had equal contribution.

Benchmark Study for DFT Calculations

For the benchmarking study of the DFT method to be used for the calculations of thermodynamic properties of γ-CD-MOF, we have optimized the cluster model generated from crystal structure of γ-CD-MOF (LAJLAL) by using B3LYP/6-31+G(d), B3LYP-D2/6-31+G(d), PBE/6-31+G(d), PBE-D2/6-31+G(d) in conjunction with both implicit CPCM and SMD solvation models, where water was used as the solvent.

The selected bond lengths ($r_{K—O}$, $r_{C—O}$ in glycosidic bonds and in glucose rings) were compared with the respective bond lengths in crystal structure of γ-CD-MOF. According to the results given in **Table S1**, K—O coordination bond distances ($r_{K—O}$) are observed to be underestimated when SMD solvation model was employed, independent from the functional used. On the other hand, B3LYP and PBE functionals, when employed without dispersion correction, are found to give slightly larger $r_{K—O}$ values than the corresponding bond lengths in the crystal structure, whereas B3LYP-D2 and PBE-D2 functionals provide close $r_{K—O}$ values. Considering $r_{C—O}$ values, all methodologies give similar bond lengths in the range of 1.40-1.46 Å, in the limits of experimentally observed distances (1.30-1.54 Å). Both B3LYP-D2 and PBE-D2 functionals in conjunction with CPCM solvation model satisfy the general trends in experimental bond lengths, thus, the results presented in this study are reported based on the B3LYP-D2/6-31+G(d) // CPCM calculations.

Table S1. Experimental (LAJLAL) and calculated bond lengths (maximum-minimum, Å) for optimized geometries of γ-CD-MOF cluster model with respect to the different DFT functionals and solvation models, using 6-31+G(d) basis set.

Bond Type	LAJLAL	B3LYP CPCM	B3LYP SMD	B3LYP-D2 CPCM	B3LYP-D2 SMD
$r_{K—O}$	2.79-2.95	2.89-3.02	2.72-2.78	2.86-2.89	2.72-2.73
$r_{C—O}$ (glycosidic)	1.39-1.54	1.41-1.44	1.41-1.45	1.40-1.44	1.41-1.44
$r_{C—O}$ (glucose)	1.30-1.53	1.40-1.44	1.41-1.45	1.40-1.45	1.41-1.45
**Bond Type**	**LAJLAL**	**PBE CPCM**	**PBE SMD**	**PBE-D2 CPCM**	**PBE-D2 SMD**
$r_{K—O}$	2.79-2.95	2.92-3.03	2.75-2.79	2.89-2.92	2.73-2.75
$r_{C—O}$ (glycosidic)	1.39-1.54	1.42-1.45	1.42-1.45	1.41-1.45	1.42-1.45
$r_{C—O}$ (glucose)	1.30-1.53	1.41-1.46	1.42-1.46	1.41-1.46	1.41-1.45

Table S2. The calculated yields of γ-CD-MOFs.

Sample Name	% Yield
**1A, 1B, 1C**	68, 84, 76
**2A, 2B, 2C**	85, 74, 57
**3A, 3B, 3C**	14, 17, 57
**4A, 4B, 4C**	84,81, 80
**5A, 5B, 5C**	67, 68, 73

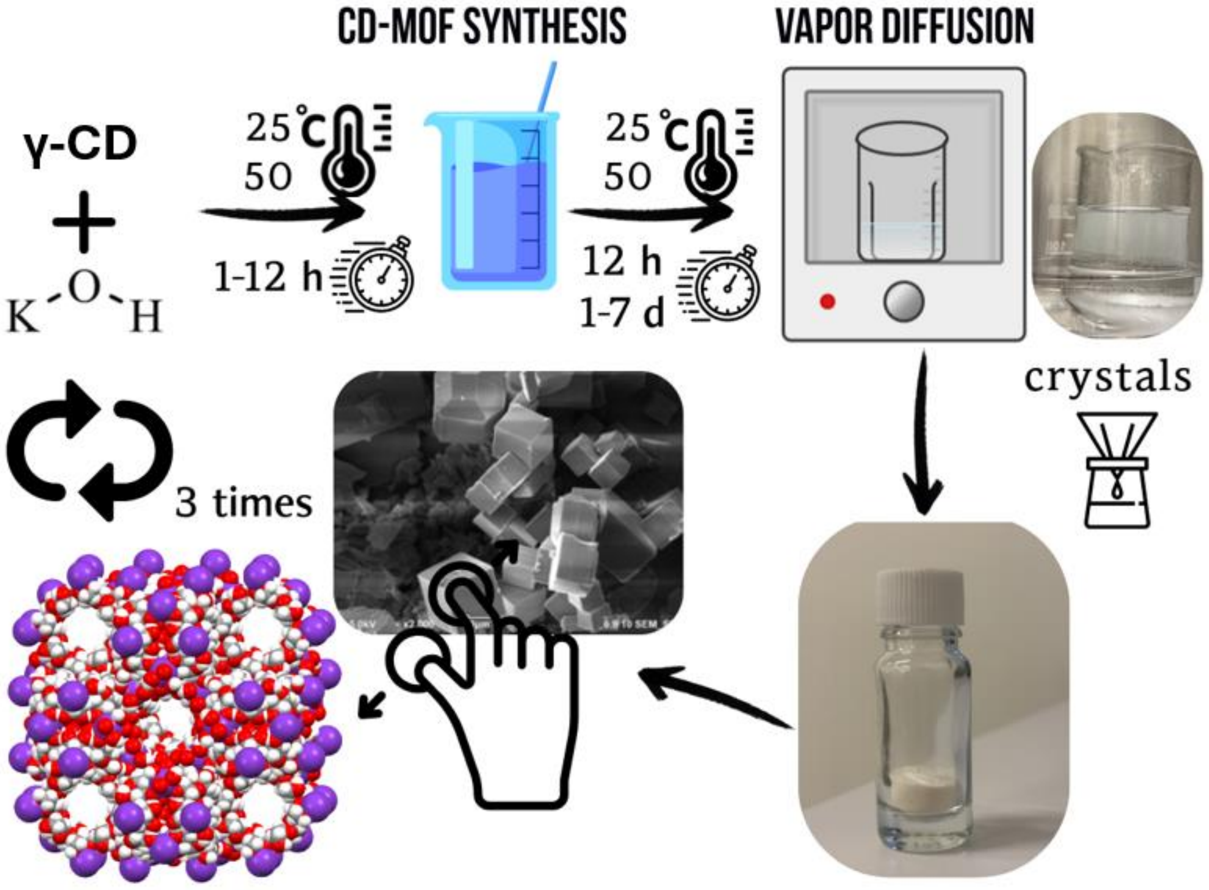

Figure S1. The schematic representation of the synthesis of γ-CD-MOF.

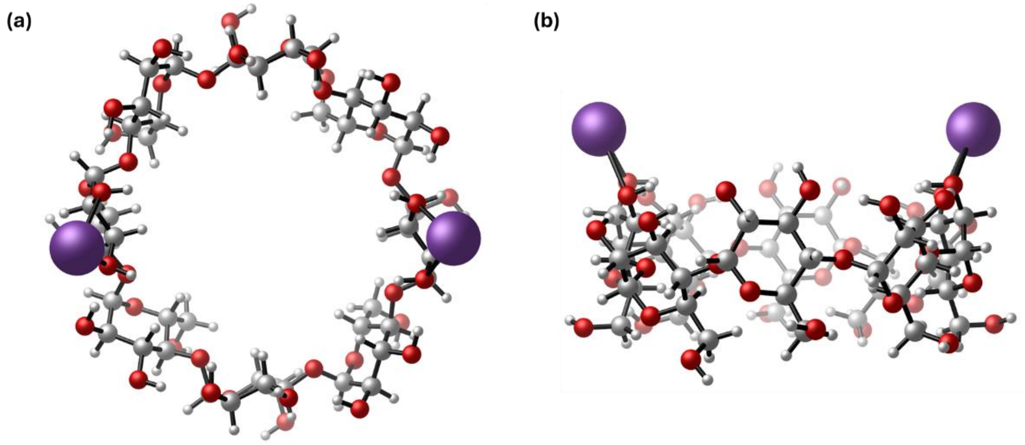

Figure S2. 3D representation of generated cluster model of γ-CD-MOF from top (a) and side (b) views. Color scheme: K = purple, O = red, C = gray, H = white.

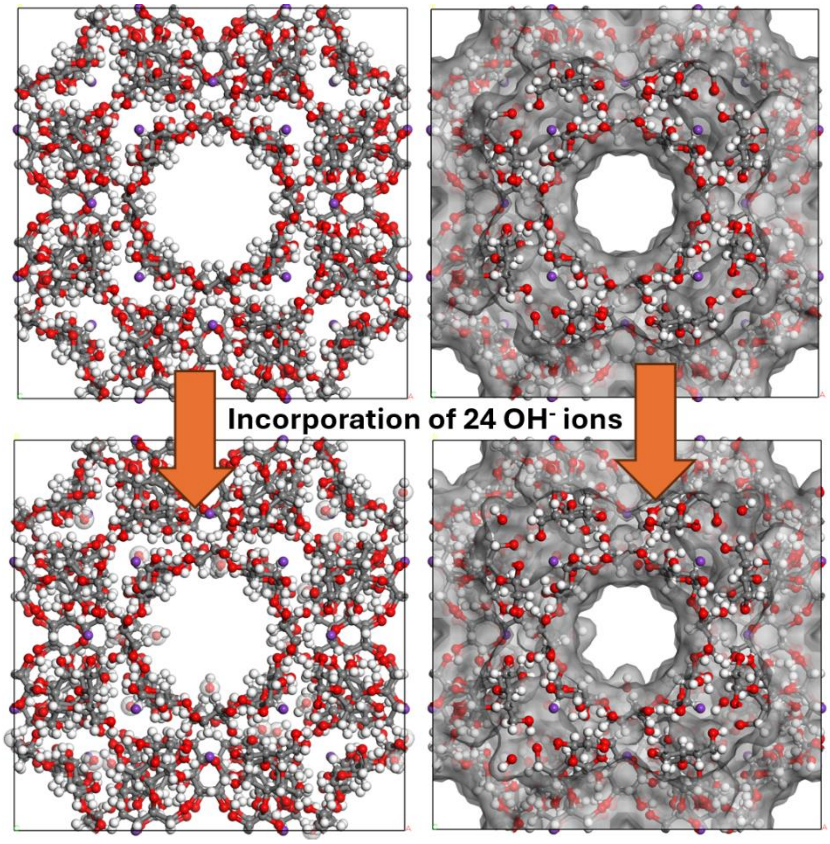

Figure S3: Atomic structure of γ-CD-MOF. The upper panels show the cationic framework, while the lower panels display the charge-neutral structure after incorporation of 24 OH^- ions. Color scheme: K = purple, O = red, C = gray, H = white.

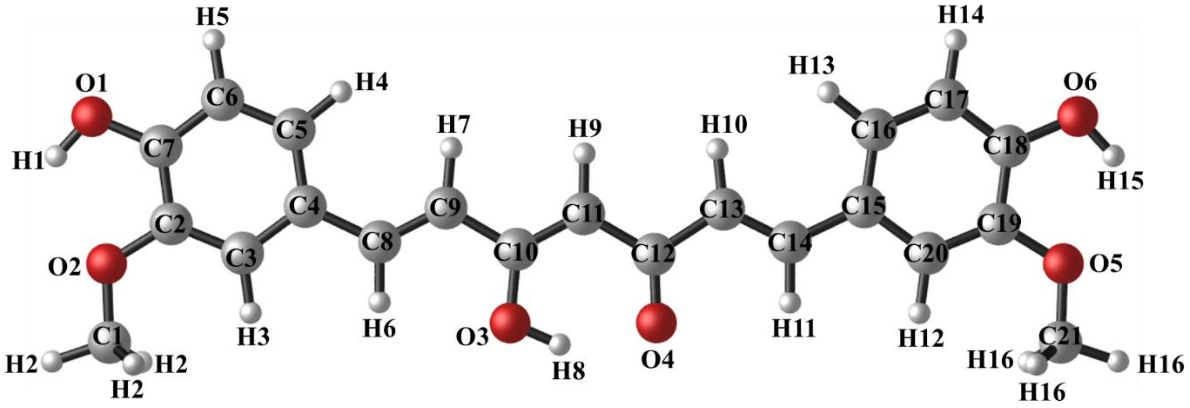

Atom	RESP Charge	Atom	RESP Charge	Atom	RESP Charge	Atom	RESP Charge	Atom	RESP Charge
C1	-0.097	C10	0.697	C19	0.180	H1	0.412	H10	0.187
C2	0.195	C11	-0.849	C20	-0.289	H2	0.097	H11	0.141
C3	-0.279	C12	0.863	C21	-0.096	H3	0.167	H12	0.175
C4	0.024	C13	-0.456	O1	-0.575	H4	0.167	H13	0.169
C5	-0.187	C14	-0.018	O2	-0.292	H5	0.199	H14	0.201
C6	-0.269	C15	0.073	O3	-0.665	H6	0.147	H15	0.411
C7	0.286	C16	-0.201	O4	-0.675	H7	0.186	H16	0.096
C8	0.013	C17	-0.273	O5	-0.288	H8	0.505		
C9	-0.411	C18	0.298	O6	-0.576	H9	0.222		

Figure S4. 2D representation of enol form of curcumin molecule and the calculated RESP charges for each atom. Color scheme: O = red, C = gray, H = white.

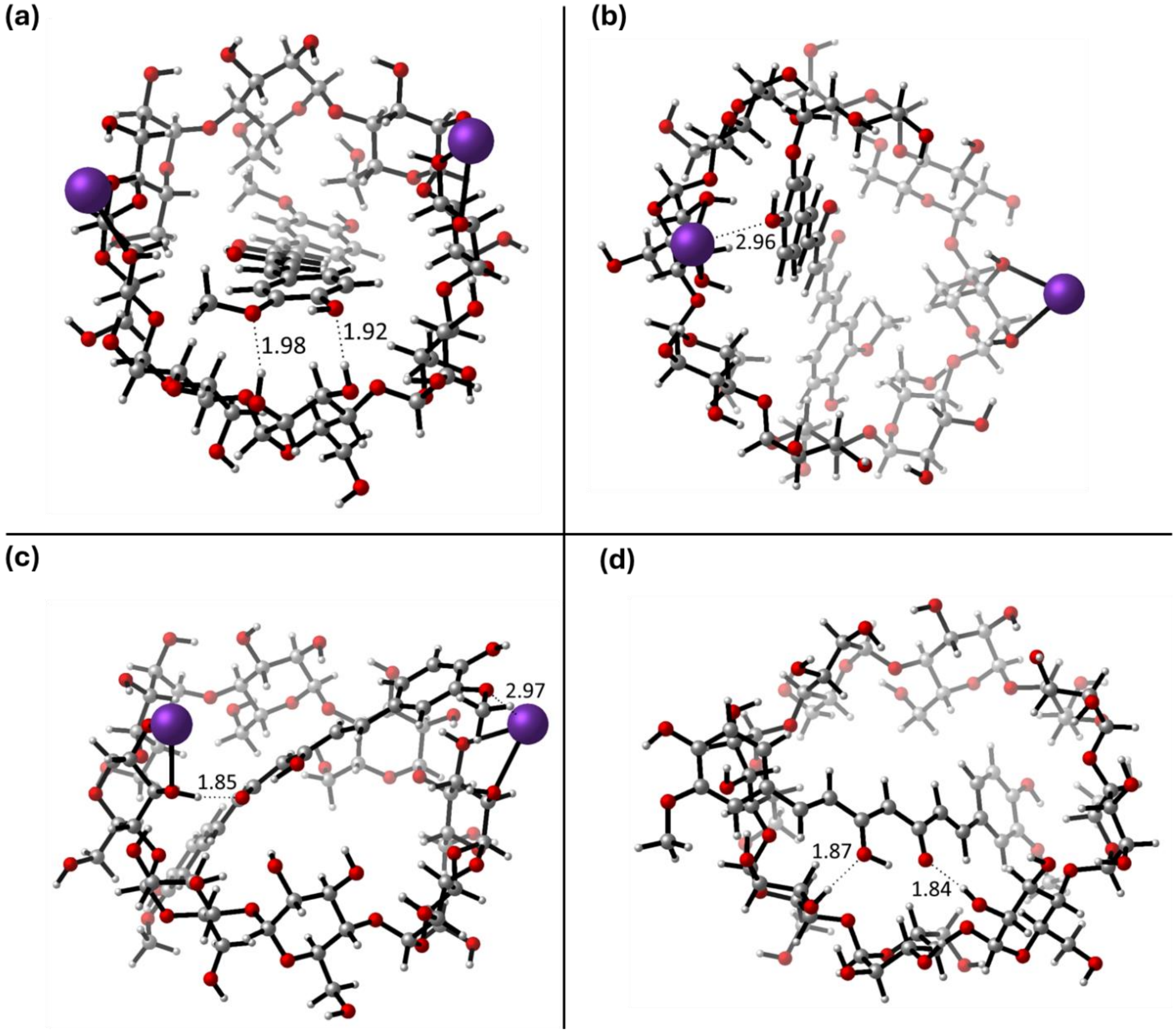

Figure S5. Three-dimensional representations of the optimized structures of γ-CD-MOF and γ-CD models in the presence of curcumin: **(a)** conformer 1 for γ-CD-MOF–curcumin, **(b)** conformer 2 for γ-CD-MOF–curcumin, **(c)** conformer 3 for γ-CD-MOF–curcumin and **(d)** γ-CD–curcumin complex. Selected intermolecular bond distances are given in Å. Color scheme: K = purple, O = red, C = gray, H = white.

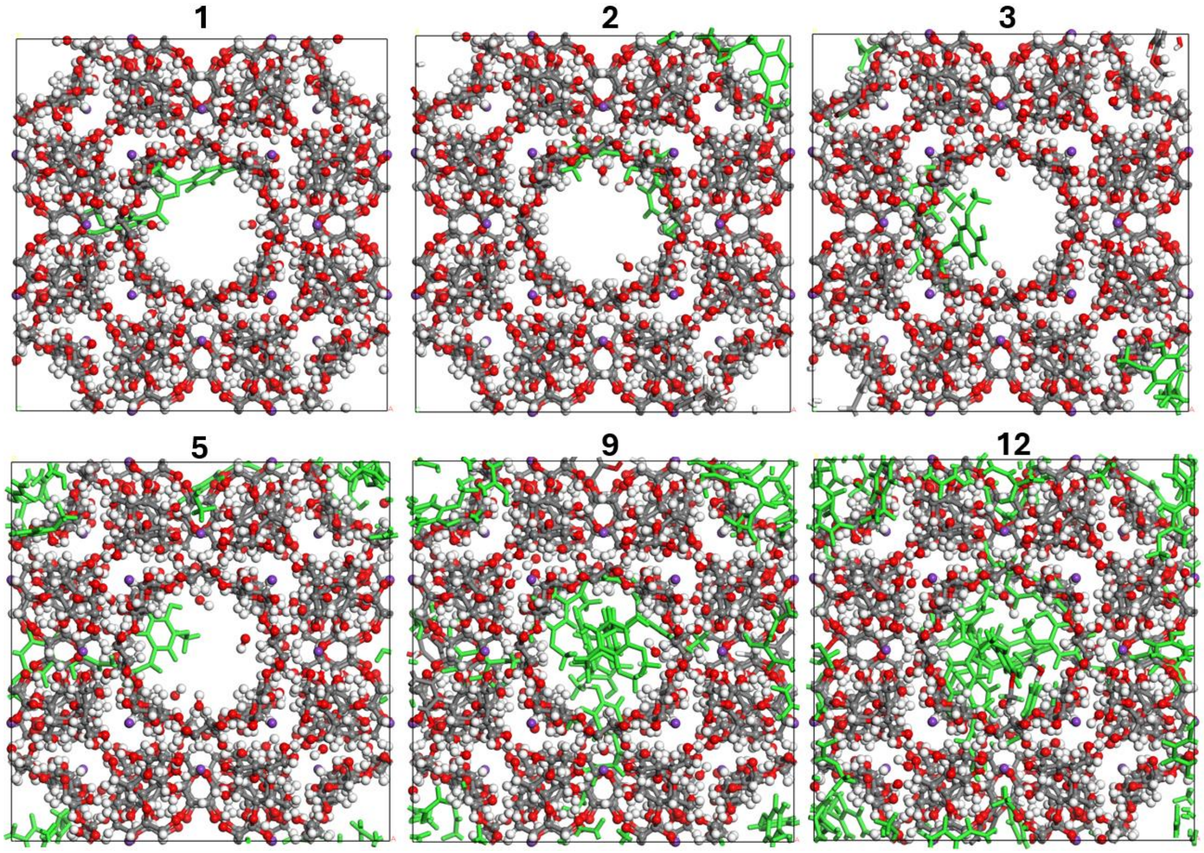

Figure S6. Atomic structure of curcumin-loaded (1, 2, 3, 5, 9 and 12 molecules) γ-CD-MOFs. Color scheme: K = purple, O = red, C = gray, H = white, curcumin = green.

Table S3. Cartesian coordinates of γ-CD-MOF cluster model optimized at B3LYP-D2/6-31+G(d) level of theory employing CPCM implicit solvation model for methanol.

O	-2.11516	6.61965	-1.20568
C	-1.32351	6.79175	-0.03604
H	-0.86887	7.78008	-0.14349
O	-1.34941	6.78909	2.40148
H	-0.90101	5.92805	2.47909
C	-2.18052	6.73968	1.23951
H	-2.79971	7.63987	1.27055
O	-4.04840	5.59756	2.31778
H	-3.57947	5.60312	3.16839
C	-3.08793	5.50842	1.26424
H	-2.46940	4.60878	1.39785
O	-4.50163	4.09777	0.00815
C	-3.85249	5.36262	-0.04435
H	-4.58484	6.17250	-0.13984
C	-2.88617	5.39396	-1.23408
H	-2.21017	4.53440	-1.15721
O	-4.53745	6.41621	-2.72916
H	-4.05273	7.25681	-2.67822
C	-3.59138	5.35689	-2.58164
H	-4.15244	4.42343	-2.66223
H	-2.83660	5.39186	-3.37933
O	-5.87326	3.07064	-1.56223
C	-5.82886	3.97849	-0.48166
H	-6.18297	4.93538	-0.87376
O	-6.64007	4.44520	1.76259
H	-5.69988	4.63620	1.97038
C	-6.70525	3.51140	0.69701
H	-7.74841	3.49176	0.36438
O	-7.25360	1.60083	2.05205
H	-6.97750	0.70899	2.33225
C	-6.29865	2.09367	1.11184
H	-5.29895	2.12541	1.56683
O	-0.33838	5.79457	0.06646
C	-6.24855	1.18645	-0.11793
H	-7.26554	1.06246	-0.50702
C	-5.37194	1.75877	-1.23147
H	-4.33655	1.85185	-0.88137
O	-6.63718	0.37663	-2.86840
H	-6.79842	-0.41106	-2.31312
C	-5.36428	0.90612	-2.50180
H	-5.02400	1.53349	-3.33145
H	-4.63768	0.09665	-2.36784

O	2.23231	-6.72669	-0.99148
C	1.29293	-6.69770	0.06389
H	0.82287	-7.68404	0.06388
O	1.00577	-6.29481	2.45612
H	0.57586	-5.42881	2.33613
C	1.98155	-6.43448	1.41514
H	2.57850	-7.31184	1.67636
O	3.73082	-5.12739	2.51125
H	3.17342	-4.98569	3.29364
C	2.89420	-5.20697	1.35417
H	2.27436	-4.30172	1.27971
O	4.46072	-3.99787	0.05656
C	3.80383	-5.26000	0.13461
H	4.53456	-6.06707	0.26194
C	2.98771	-5.50941	-1.14006
H	2.30474	-4.66540	-1.30017
O	4.82274	-6.72047	-2.22851
H	4.34005	-7.54850	-2.06822
C	3.85554	-5.67865	-2.37803
H	4.41526	-4.75683	-2.55231
H	3.20390	-5.87250	-3.24081
O	5.99087	-3.15309	-1.46300
C	5.82661	-3.97736	-0.33071
H	6.15538	-4.97493	-0.63341
O	6.45677	-4.32669	1.98735
H	5.49602	-4.40397	2.17262
C	6.64908	-3.47272	0.86994
H	7.71137	-3.53427	0.61116
O	7.20431	-1.50899	2.14252
H	6.95248	-0.58936	2.35308
C	6.29484	-2.01345	1.16453
H	5.26480	-1.97313	1.54659
O	0.32921	-5.68479	-0.11918
C	6.36534	-1.17303	-0.11250
H	7.41250	-1.06414	-0.41678
C	5.57521	-1.78634	-1.27105
H	4.50232	-1.75427	-1.04482
O	7.20044	-1.11671	-2.98185
H	7.45717	-2.05192	-3.04592
C	5.82599	-1.07886	-2.59666
H	5.19334	-1.53729	-3.36887
H	5.56100	-0.02467	-2.49594
O	6.67364	1.94335	-0.97906
C	6.67733	1.21388	0.23204
H	7.71071	0.88644	0.37251

O	6.16729	1.30737	2.61766
H	5.33179	0.80817	2.59957
C	6.23408	2.09101	1.42016
H	7.00765	2.84478	1.59247
O	4.60710	3.71626	2.17427
H	3.67907	3.60584	2.43496
C	4.90498	2.78060	1.12015
H	4.11022	2.02660	1.06888
O	3.68528	4.02750	-0.48174
C	4.98059	3.50032	-0.22225
H	5.72094	4.30555	-0.16249
C	5.39593	2.51506	-1.32277
H	4.64115	1.72149	-1.40098
O	6.48351	4.26090	-2.65875
H	7.34453	3.90999	-2.37579
C	5.55990	3.17189	-2.68493
H	4.59795	3.58401	-3.00073
H	5.87510	2.40980	-3.41032
O	2.61364	5.31262	-2.06593
C	3.62364	5.31956	-1.08833
H	4.55933	5.53520	-1.61062
O	4.39350	6.35686	0.98009
H	4.40712	5.47882	1.41595
C	3.36341	6.37835	0.00240
H	3.41153	7.36593	-0.46824
O	1.67647	7.30127	1.44246
H	0.77713	7.19006	1.80214
C	1.96500	6.19048	0.59546
H	1.93737	5.25456	1.17235
O	5.80183	0.11427	0.18752
C	0.93571	6.09154	-0.53034
H	0.88751	7.05428	-1.05179
C	1.29950	5.01026	-1.54808
H	1.32554	4.02870	-1.05914
O	-0.09757	6.18690	-3.23516
H	-0.81564	6.50797	-2.65463
C	0.32717	4.92451	-2.72581
H	0.82836	4.39643	-3.54296
H	-0.53688	4.32665	-2.41497
O	-6.58361	-1.84695	-0.97868
C	-6.62102	-1.16875	0.27092
H	-7.65350	-0.82699	0.37899
O	-6.21257	-1.34814	2.66102
H	-5.39307	-0.82247	2.66564
C	-6.24561	-2.09452	1.44237

H	-7.04342	-2.83340	1.55952
O	-4.67571	-3.77040	2.21192
H	-3.76797	-3.65200	2.53300
C	-4.92341	-2.80974	1.17022
H	-4.11017	-2.07442	1.15099
O	-3.70856	-4.07426	-0.42476
C	-4.98683	-3.50309	-0.18715
H	-5.76111	-4.27842	-0.16559
C	-5.31667	-2.47991	-1.28086
H	-4.52434	-1.72241	-1.30394
O	-6.42041	-4.14761	-2.69932
H	-7.28376	-3.76650	-2.46769
C	-5.45421	-3.09763	-2.66418
H	-4.49712	-3.54171	-2.94879
H	-5.70651	-2.30726	-3.38425
O	-2.72912	-5.32637	-2.10109
C	-3.67611	-5.35203	-1.06559
H	-4.64008	-5.56108	-1.53603
O	-4.33704	-6.41726	1.01690
H	-4.36893	-5.53052	1.43314
C	-3.34888	-6.41930	-0.00405
H	-3.40369	-7.40416	-0.47962
O	-1.57859	-7.28443	1.38128
H	-0.73913	-7.06116	1.83071
C	-1.92956	-6.20068	0.52415
H	-1.90251	-5.25337	1.08152
O	-5.72505	-0.08850	0.29446
C	-0.94359	-6.08884	-0.64407
H	-0.85294	-7.05972	-1.14284
C	-1.38459	-5.03864	-1.66879
H	-1.35901	-4.04585	-1.20206
O	-0.44556	-6.28343	-3.50854
H	0.23086	-6.26131	-4.20259
C	-0.49702	-4.99135	-2.89809
H	-0.90245	-4.24703	-3.59696
H	0.50232	-4.67222	-2.57899
K	-6.15677	-3.39125	4.67033
K	6.03728	3.32087	4.64288
